# Autotransplantation of a Third Molar to Replace Compromised Molar With the Individual Three-Dimensional Printed Ultrasonic Osteotome: A Case Report

**DOI:** 10.1155/crid/6146337

**Published:** 2025-01-20

**Authors:** Cheng Bi, Wen Zhang, Bin Peng

**Affiliations:** ^1^Department of Endodontics, Hangzhou Stomatology Hospital, Hangzhou, Zhejiang, China; ^2^Sadlon Centre for Health, Wellness and Sciences, Georgian College, Barrie, Ontario, Canada

**Keywords:** case report, piezosurgery, three-dimensional printing technique, tooth autotransplantation, tooth root–shaped ultrasonic osteotome

## Abstract

Tooth autotransplantation is widely used to replace congenitally missing teeth or teeth with irreversible damage. This case report presents a personalized ultrasonic osteotome that enables precise preparation, minimizes bone trauma, enhances the initial stability of the transplanted tooth, and contributes to a favorable prognosis. The procedure is as follows: a 25-year-old female patient presented with a porcelain-fused-to-metal crown on Tooth #19, which had detached due to severe decay, rendering the tooth unsalvageable. Tooth #32 exhibited mesial inclination, resulting in decay of Tooth #31. After presenting treatment plan, the patient provided informed consent by signing the necessary documentation to proceed with the autotransplantation of Tooth #32 to replace Tooth #19. Tooth #32 was extracted and immediately scanned chairside using a 3Shape scanner. A custom-designed, tooth root–shaped ultrasonic osteotome was fabricated using 3D printing technology to match the shape of Tooth #32. Tooth #19 was extracted using a minimally invasive technique. The alveolar socket of Tooth #19 was prepared using the customized 3D-printed ultrasonic osteotome and a computer-aided rapid prototyping resin model. Tooth #32 was then transplanted into the socket and secured with figure-eight suspension sutures. To prevent damage to bone cells and periodontal tissues from overheating during socket preparation, the ultrasonic osteotome was designed with a porous, water-cooled system, effectively addressing this issue. After 24 months of follow-up, the transplanted tooth met success criteria, with no signs of pathological radiolucency, root resorption, or pain in the donor tooth. The alveolar socket, prepared with the personalized ultrasonic osteotome, ensured an optimal fit for the donor tooth, maintaining its stability and minimizing postoperative complications. The use of 3D printing technology to create a personalized ultrasonic osteotome represents an innovative advancement in tooth transplantation, supporting the development of digital and minimally invasive techniques in this field.

## 1. Introduction

Tooth autotransplantation is to replace an erupted/unerupted tooth from one site to another in the same patient [1–3]. It is safe, reliable, and widely used to replace congenitally missing teeth or teeth with irreversible damage. The survival rate of tooth autotransplantation varies from 84% to 100% [1, 4].

Although transplantation of teeth with immature roots can improve pulpal vascularization and vitality [3], utilizing fully developed teeth as donors is a more commonly utilized approach in adult patients [2, 5]. Transplanting fully developed teeth has a positive impact on preventing periodontal or pulp-related diseases [5, 6]. Using this method can lower the chances of root resorption and improve success rate.

A few clinical reports have used mature third molars as donors for autotransplantation. According to the research conducted by Yu and Xia, the transplantation of fully developed third molars into recently extracted sockets resulted in a survival rate of up to 93.1% over 10 years [7, 8]. Numerous studies have indicated that transplanting a third molar is a superior option to dental implants or fixed partial prostheses [2, 7, 8]. This is due to its ability to withstand occlusal loading and preserve periodontal ligament (PDL) and surrounding alveolar bone.

The survival of transplanted tooth mainly depends on the viability of PDL cells attached to donor tooth. Thus, it is crucial to extract and handle donor tooth gently during transplantation, without causing any damage to PDL. Manipulating tooth during surgery and extending time it is outside of the mouth can negatively impact PDL, resulting in postoperative issues like gradual root deterioration and reduced attachment [1, 5, 9]. Therefore, simplifying surgery steps and decreasing the time of procedure are crucial points to maximize the success of tooth transplantation.

The utilization of 3D design and printing technology is becoming common in tooth autotransplantation [7, 10, 11]. This is aimed at improving success rates and facilitating better periodontal healing. A widely accepted method is the computer-aided rapid prototyping (CARP) model. It creates 3D replicas of donor tooth, resulting in less exposure of donor tooth outside of the mouth and better matching between donor tooth and recipient site [7, 12]. In 2016, Moin et al. developed an innovative method for tooth transplantation which used a custom-made surgical osteotome, designed with 3D printing technology [10]. The custom-printed osteotome with tapping technique allows quick and minimally invasive preparation of the alveolar socket on a cadaver. In 2022, a 9-year-old boy received a treatment based on the upon technique [11]. His first premolar was conducted to replace a maxillary ankylosed incisor by utilizing a custom-designed osteotome with tapping technique. However, the osteotomes developed by Moin and Sans were only capable of making tapered alveolar sockets with the tapping technique and might not be suitable for preparing more complicated sockets.

Thanks to the latest developments in additive manufacturing and ultrasonic technologies, it is now feasible to produce a personalized ultrasonic osteotome in root shape. This breakthrough innovation has the potential to significantly enhance the success rate of tooth autotransplantation by minimizing the distance between new alveolus and donor tooth. This case report is aimed at introducing a 3D-printed ultrasonic osteotome that offers several impressive features in tooth autotransplantation. The root-shaped ultrasonic osteotome is user-friendly due to its small vibration amplitude and can prepare intricate alveolar sockets, making it highly beneficial for tooth autotransplantation purposes.

## 2. Case Report

### 2.1. Ethics

Before conducting oral examinations in a clinical setting, the subject provided informed consent. This was approved by Hangzhou Stomatology Hospital Authority for ethical purposes (Approval No. 2021LL06).

### 2.2. Clinical Examination and Treatment Planning

A 25-year-old woman had a porcelain-fused-to-metal crown on her left lower first molar (Tooth #19, universal numbering system) fallen off, causing difficulty with chewing. She promptly went to the Hangzhou Stomatology Hospital for assessment and treatment. Before conducting a thorough oral and dental treatment, the patient's medical and dental records were reviewed.

**Figure 1:**
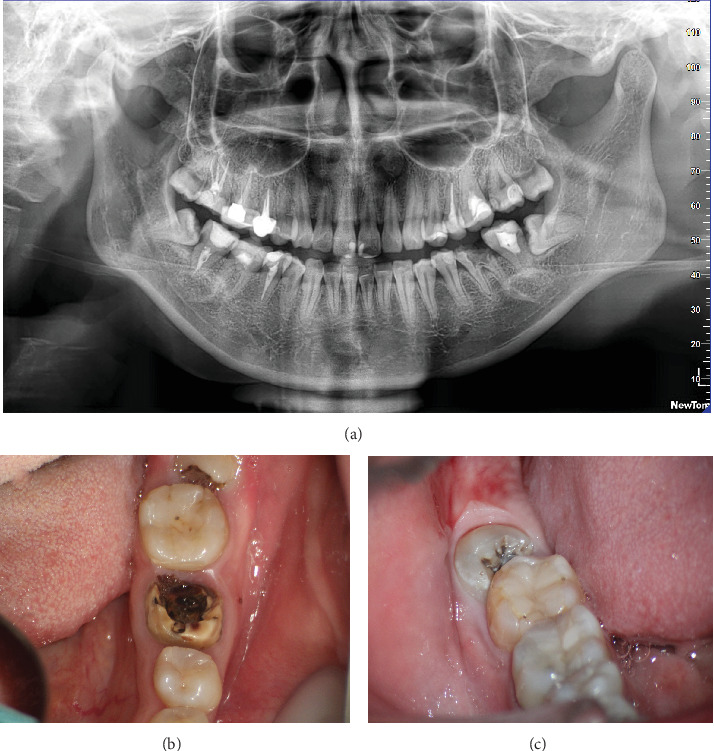
Preoperative panoramic radiograph and photographs. (a) Panoramic radiograph shows an unrestorable Tooth #19. The root of Tooth #32 is fused, making minimally invasive extraction available. (b) The preoperative clinical picture of Tooth #19. (c) The preoperative clinical picture of Tooth #32.

A pretreatment panoramic radiograph was obtained (Figure 1(a)). The panoramic radiograph revealed a small amount of bone resorption at the root bifurcation of Tooth #19. The root of Tooth #32 was fused, making minimally invasive extraction available. Moreover, Tooth #32 was close to the inferior alveolar nerve and had a single root, while Tooth #31 had a composite resin on its occlusal surface and caries on the distal surface. The panoramic radiograph also showed that the root canals of Tooth #31 were filled, and there were no visible issues in its apical area. Clinical photos presented that Tooth #19 had a severe defect in its crown, with a large area of caries reaching the pulp cavity. The remaining crown was weak, especially at the root bifurcation and distal part of the crown (Figure 1(b)). Tooth #32 on the right mandible had caries on the occlusal surface with mesial inclination, but there was no redness or swelling of the gums (Figure 1(c)). Because Tooth #19 had a severe crown defect and root bifurcation lesion, it was recommended for extraction. Tooth #32 caused caries in Tooth #31 due to its mesial inclination, so it was also recommended for extraction to prevent further decay. We thoroughly explained various possible treatment options to the patient, including a single-tooth implant, removable denture, and autotransplantation of tooth. The patient expressed strong interest in transplanting Tooth #32 to Tooth #19 site. After analyzing factors such as cost, treatment duration, efficacy, and potential complications, the patient ultimately decided to opt for autotransplantation to preserve her natural tooth as much as possible. We also informed the patient of the associated risks and the potential remedial options in case of transplant failure, such as a single-tooth implant. The patient understood and signed a consent form to proceed with the autotransplantation plan.

### 2.3. Clinical Procedures

**Figure 2:**
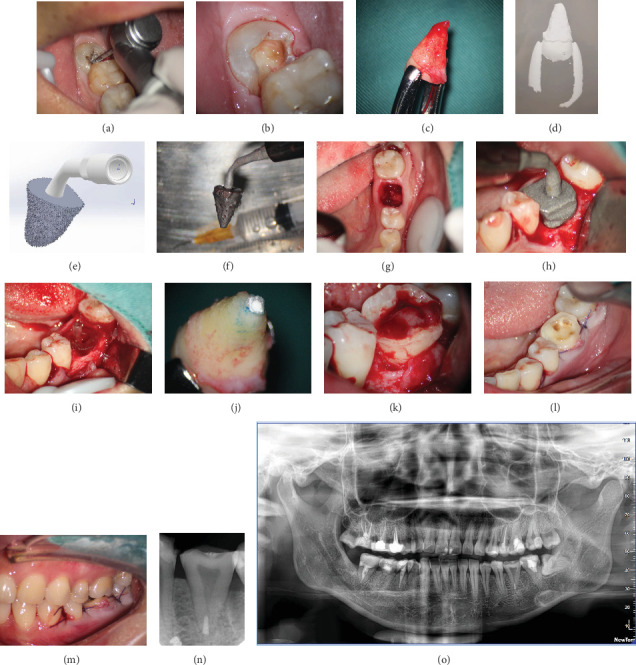
The clinical procedure. (a) Removal of dental caries on the mesial–occlusal part of Tooth #32 using a fissure bur. (b) Tooth #32 after removal of carious tissue. (c) Tooth #32 was extracted with a minimally invasive technique. (d) Tooth #32 was scanned digitally by a 3Shape scanner to obtain a highly accurate 3D image. (e) A custom 3D ultrasonic osteotome was designed. (f) A 3D-printed ultrasonic osteotome. (g) Occlusal view of recipient alveolar socket. (h) A 3D-printed individual ultrasonic osteotome was used to prepare the recipient's alveolar socket. (i) A CARP model was used to evaluate and prepare the receptor alveolus. (j) The root end of Tooth #32 was prepared and filled with iRoot BP. (k) The prepared Tooth #32 was transplanted into the recipient site. (l) Occlusal view of the transplanted tooth with suture 1 week after surgery. (m) Buccal view of the transplanted tooth with suture 1 week after surgery. (n) Periapical radiograph of the transplanted tooth 1 week after surgery. (o) Panoramic radiograph of the transplanted tooth 1 week after surgery.

Before extraction of Tooth #32, caries on the tooth's occlusal surface were removed to minimize bacterial contamination and postoperative discomfort (Figure 2(a)). The operation on Tooth #32 was performed without the use of a dental dam. This decision was made due to the posterior position of the tooth and the patient's significant gag reflex, which made the placement of a dental dam impractical. Caries was removed from the mesial–occlusal surfaces of Tooth #32 and the distal–occlusal surfaces of Tooth #31 (Figure 2(b)). Subsequently, the prepared cavities on Teeth #32 and #31 were restored using composite resin.

Before the extraction of #32, the patient received antibiotic prophylaxis (amoxicillin/clavulanic acid, 500/125 mg) 1 h prior. The surgery was performed by the same experienced dentist under local anesthesia (lidocaine 2% with epinephrine 1:100,000). Briefly, using maxillary premolar extraction forceps (Köhler, Mainz, Germany), gently separate the roots of the donor tooth from the surrounding bone tissue and carefully remove it from the alveolar bone to avoid damaging surrounding structures (Figure 2(c)). The extraction process also ensured that periodontal membrane of the tooth was not affected. After extraction, it was observed that there was uniform coverage of soft tissue around the tooth root. The dentist held the tooth using premolar extraction forceps. A chairside dental assistant used an R700 scanner (3Shape, Copenhagen, Denmark) to create 3D digital images of the tooth's shape (Figure 2(d)). A sterilized scan tip was applied to prevent infection during scanning. The scanning procedure was very quick, taking only 10–15 s. Due to the presence of blood and periodontal membrane on the tooth root surface, the final personalized ultrasonic osteotome (Figure 2(e)) was designed to be about 1 mm larger than the tooth root. This extra space allowed for the growth of periodontal membrane cells, even if some minor damage occurred during the extraction process. This damage could be easily repaired and did not cause root resorption. In the current case report, we directly utilized the 3Shape scanner to capture 3D images of the tooth for higher accuracy compared to the traditional cone-beam computed tomography (CBCT) capture method. Implementing this strategy ensures a reduction of artificial biases in obtaining images.

We informed the dental laboratory about the dental surgery 1 day before the clinical operation, ensuring they were well prepared. After scanning, Tooth #32 was delicately repositioned into its original alveolar socket to maintain periodontal membrane. The scan data was sent to the dental laboratory in Hangzhou Stomatology Hospital, where a tooth root–shaped ultrasonic osteotome tip was swiftly produced using the titanium printer (iSLM280, Zrapid Technologies Co. Ltd., Suzhou, China). Since the scan data was obtained using a 3Shape scanner, it was directly input into computer software for processing. The images of the tooth extraction forceps were removed in the software, allowing us to construct the ultrasonic osteotome smoothly. To prevent damage to local tissues and cells during alveolar socket preparation, the root-shaped osteotome had multiple water-cooled vents on its surface (Figure 2(f)). It was also designed with rough emery surface to improve the preparation efficiency. It took totally about 15 min to complete the scanning and printing process. Before clinical use, the ultrasonic osteotome was disinfected by 5% povidone-iodine solution for 10 min.

**Figure 3:**
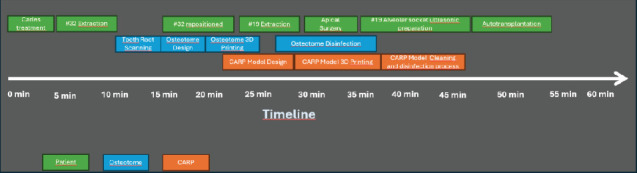
Timeline of the tooth autotransplantation procedure.

During the extraction of Tooth #19, we used a minimally invasive approach without a mucoperiosteal flap surgery. Additionally, we performed a root-splitting procedure without using tooth elevator or removing alveolar compartment (Figure 2(g)). The alveoli were visually inspected and confirmed that the buccal cortical plate remained undamaged. Afterward, the 3D-printed osteotome in the shape of Tooth #32 was positioned at the location where it was needed (Figure 2(h)). In order to improve efficiency of the autotransplantation, we also used a 3D printer (Form2; Formlabs, Somerville, Massachusetts) to create a CARP model (Figure 2(i)) of donor Tooth #32. The model had a 50-mm stacking pitch (8). To ensure safety, harmless resin (D031, Hangzhou Leyi New Material Technology Co. Ltd., Hangzhou, China) was used in the CARP model. Before clinical use, the CARP was disinfected in 5% povidone-iodine for 10 min. The donor Tooth #32 received an ex vivo apical surgery, and its apical part was sealed with iRoot BP (Innovative Bioceramix in Burnaby, Canada) (Figure 2(j)). The apicoectomy and retrofill procedure took about 5 min. The use of a personalized ultrasonic working tip ensured precise matching between the prepared alveolar socket and the donor tooth. In this case report, we prepared only the mesial alveolar socket of the mandibular first molar, which contained cancellous bone, using the individual osteotome with Ultrasurgery System (Guilin Woodpecker Medical Instrument Co. Ltd., China) and 250 mL of 10°C saline. The preparation time was short, taking approximately 1 min. Afterward, the donor tooth was replaced in the new location carefully. The PDL was positioned about 1–2 mm above the alveolar ridge (Figure 2(k)). To prevent crown displacement, a figure-eight suspension suture was employed: the suture needle was inserted at the mesial buccal gingiva of the transplanted tooth, passed obliquely through the occlusal surface to the distal lingual gingiva, then through the mesial lingual gingiva, and crossed over the occlusal surface to enter the distal buccal gingiva (Figures 2(l) and 2(m)). The entire process of extracting donor tooth, endodontic surgery, and transplantation took about 45–50 min by the same dentist. Donor tooth stability was successfully achieved after transplantation (Figures 2(n) and 2(o)). Postoperatively, the patient was prescribed 500 mg of amoxicillin every 8 h for a week and 600 mg of ibuprofen every 8 h for 3 days. A soft diet was recommended for 2 weeks. Six months later, a Lava all-ceramic crown was provided to restore and protect the tooth. The timeline of the tooth autotransplantation procedure is shown in the figure to enhance the readers' understanding (Figure 3).

### 2.4. Follow-Up Examinations

**Figure 4:**
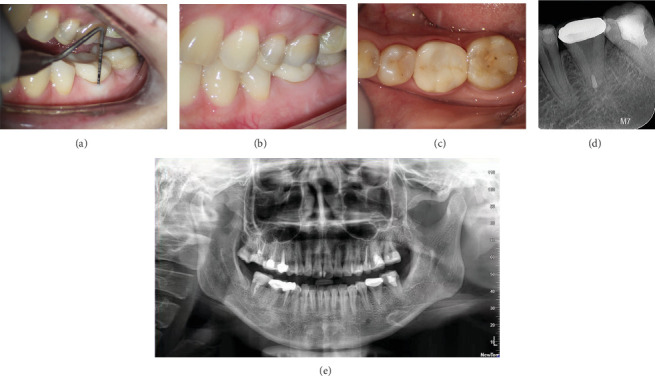
Clinical follow-up at 2 years. (a) The periodontal probing depth of the transplanted tooth was normal, which meant that tooth autotransplantation obtained excellent epithelial attachment. (b) The buccal view of the transplanted tooth. (c) The occlusal view of the transplanted tooth. (d) Periapical radiograph 2 years after transplantation. (e) Panoramic radiograph 2 years after transplantation.

Follow-up examinations at 1 week, 1 month, 6 months, 1 year, and 2 years posttransplant revealed no pain or other periapical pathologies associated with the transplanted tooth (Figures 4(a)–4(e)). The autotransplanted tooth with Lava crown did not show any signs of root decay. The transplantation was deemed successful according to previous criteria [1, 6].

## 3. Discussion

The primary innovation of this study lies in the use of 3D printing technology to create personalized ultrasonic osteotome and apply it to autogenous tooth transplantation. This approach combines personalized 3D scanning and printing technology with minimally invasive alveolar bone preparation using an ultrasonic osteotome. In the current case report, we directly utilized the 3Shape scanner to capture 3D images of the tooth for higher accuracy compared to the traditional CBCT capture method. Implementing this strategy ensures a reduction of artificial biases in obtaining images. By accurately replicating the morphology of the donor tooth root, it achieves minimally invasive and efficient preparation of the cancellous bone, resulting in good transplant stability in this clinical case report. Because using ultrasonic osteotomes for alveolar bone preparation poses a lower risk of damage to surrounding soft tissues (such as the inferior alveolar nerve, periosteum, Schneiderian membrane, and oral mucosa), results in less bleeding, and provides a good clinical view, they have become a commonly used tool for bone preparation in clinical practice [13, 14]. Studies have pointed out that using ultrasonic osteotomes for bone preparation during surgery also helps improve precision and reduce splintering at the bone's edge [14, 15], which is one reason why good posttransplant stability was achieved in this case report. However, it should be noted that due to the uniqueness of autogenous tooth transplantation, there are currently relatively few clinical reports on the personalized preparation of donor tooth root morphology. Most commonly used ultrasonic osteotomes are commercial products and are difficult to adapt to the morphology of the donor tooth root. Our report fills this gap, providing strong support for efficient and minimally invasive autogenous tooth transplantation.

In fact, besides the commonly seen ultrasonic osteotomes in clinical practice, bone osteotomes using physical tapping techniques for alveolar bone preparation are also encountered. This type of osteotome is widely used in orthognathic surgery, implant surgery, microsurgical apicoectomy, autogenous tooth transplantation, bone grafting, and oral cyst removal. Researchers as early as 2016 reported using 3D-printed surgical guides to guide osteotomes for alveolar socket preparation [10, 11]. This method offers several distinct advantages, such as mature 3D guide technology and fast bone preparation with minimal heat generation using physical tapping. However, objectively speaking, this technique still has some unavoidable drawbacks. Firstly, physical tapping tests the patient's psychological tolerance and requires patient cooperation. Secondly, control over physical tapping osteotomes remains challenging, making it difficult to achieve high precision. Moreover, physical tapping osteotomes cannot replicate alveolar sockets and may sometimes necessitate removing more bone tissue to match donor teeth, which can be detrimental to patients. In contrast, the approach in this case can effectively overcome these disadvantages, offering minimally invasive procedures, high precision, and good stability of transplanted teeth. In our clinical experience, its efficiency in preparing cancellous bone is appropriate. Furthermore, combining personalized ultrasonic osteotomes, 3D surgical guides, and physical tapping osteotomes could potentially yield a more rational and efficient method for alveolar bone preparation, which is a direction for future research.

In this case report, we utilized CARP, a resin-based 3D-printed tooth root model. The use of CARP during surgery was based on several considerations. Firstly, CARP plays a positive role in reducing transplantation time and minimizing PDL injury [7, 12]. Multiple studies have shown that combining precise osteotomy techniques with CARP models for preparing recipient alveolar sockets significantly reduces the time donor teeth spend outside the mouth [15, 16]. Without CARP models, preparation of recipient site alveolar sockets relies solely on donor teeth, which not only damages the donor tooth's PDL but also prolongs surgical duration. Secondly, using CARP in autogenous tooth transplantation reduces surgical complications, improves surgical precision, and enhances success rates [16, 17]. CARP models are highly reliable in assisting with alveolar socket preparation, with an average difference of only 0.29 mm between 3D-printed CARP models and donor teeth. Thirdly, CARP aids in achieving cervical closure between the root surface of the transplanted tooth and the alveolar socket of the recipient site [7]. If a closed region of alveolar bone is formed beneath the tooth's cervical area, it promotes bone healing, minimizes the risk of infections and other complications, and increases the stability of the transplanted tooth. Therefore, combining CARP with personalized ultrasonic osteotome preparation is essential and recommended.

Certainly, the use of personalized ultrasonic osteotomes also comes with some drawbacks and limitations. Studies have shown that compared to traditional mechanical osteotomy, ultrasonic tools prepare bone tissue at a slower rate. Even with increased operator experience, overall efficiency remains slower than traditional methods [14, 15]. In this case report, we only prepared the cancellous bone of the mesial alveolar socket of the mandibular first molar, thereby reducing preparation time. However, it is important to note that this method may not be suitable for all clinical scenarios. Additionally, while increasing the power of the ultrasonic osteotome can enhance cutting efficiency, it may also lead to increased heat generation. Although the ultrasonic osteotome used in this case had a water-cooled feature and a porous surface, it remains unclear whether it can withstand higher power levels and whether excessive heat production could damage the alveolar bone. Thirdly, it is worth noting that the production process of personalized 3D-printed ultrasonic osteotomes requires support from a technical team and involves significant costs. Therefore, when selecting treatment options, detailed treatment plans and alternative options should be carefully developed, understanding the characteristics of the required alveolar bone preparation and choosing the preparation tools wisely.

In this case report, the transplanted tooth did not undergo root canal treatment, based on several considerations. Firstly, the apex sealed with iRoot BP demonstrated good closure with no signs of infection. Follow-up examinations at 1 week, 1 month, 6 months, 1 year, and 2 years posttransplant revealed no pain or other periapical pathologies associated with the transplanted tooth. A clinical analysis reported that among 20 transplanted teeth that did not receive root canal treatment, 8 cases exhibited inflammation yet remained viable, while the other 12 cases were successful [18]. This suggests that the necessity of root canal treatment posttransplantation should be determined by the clinician based on thorough clinical evaluation and follow-up. In cases where the tooth remains asymptomatic and free of infection, root canal treatment may be deferred. However, it is imperative to conduct regular checkups to monitor the health of the tooth and surrounding tissues. This approach aligns with the current understanding that each case should be individually assessed to optimize clinical outcomes.

In the final part of the discussion, we aim to elucidate the rationale for utilizing fully developed third molars as donor teeth in this case report. The choice between fully developed and immature third molars for autotransplantation remains contentious. Jang et al. [19] reported successful autotransplantation of immature third molars in four patients aged 15–21 using the CARP technique. Postoperative follow-up over 2–7 years revealed that the transplanted teeth exhibited no symptoms or functional issues, with radiographic examinations showing continued root development in both length and thickness. Similarly, a case report by Rey et al. [20] demonstrated that autotransplantation using autologous leukocyte–platelet-rich fibrin (L-PRF) and immature third molars resulted in normal pulp vitality and continued root development to apex closure over a 2-year follow-up period. The authors suggested that L-PRF may enhance the vitality of pulp and PDL cells, thereby improving clinical outcomes. Gonzalez et al. [21] reported comparable results, achieving successful autotransplantation using platelet-rich plasma (PRP) and immature third molars, with PRP promoting root and nerve development. Furthermore, a systematic review and meta-analysis by Sicilia-Pasos et al. [3] analyzed 14 studies on the use of undeveloped third molars and found an overall survival rate of 97.9% for autotransplanted teeth. However, despite the potential benefits of using undeveloped third molars, such as the preservation of pulp vitality and continued root development, this approach demands high technical proficiency and careful donor tooth selection. Conversely, fully developed third molars offer a few distinct advantages, including enhanced stability and higher success rates posttransplantation [22]. These fully developed teeth better adapt to their new positions, with more favorable prognoses for root healing [23]. The integrity of the periodontal membrane is more easily maintained, facilitating the integration of the transplanted tooth into the new site [24]. In conclusion, while both fully developed and undeveloped third molars present unique advantages and limitations as donor teeth, clinical practice generally favors the use of fully developed teeth for autotransplantation due to their higher success rates, improved healing outcomes, and better functional recovery.

## 4. Conclusion

In conclusion, the introduction of personalized 3D-printed ultrasonic osteotomes presents a novel option for surgical procedures in autogenous tooth transplantation. Its attributes of minimal invasiveness and high precision contribute significantly to improving transplant tooth stability and reducing PDL damage. Furthermore, this study demonstrates its high efficiency in preparing cancellous bone within alveolar sockets. This pioneering innovation in autologous tooth transplantation technology is poised to propel the development of digitalized and minimally invasive approaches within the field, promising enhanced patient outcomes and broader application in clinical practice.

## Data Availability

Data supporting this research article are available from the corresponding author or first author upon reasonable request.
